# Blockchain-Federated and Deep-Learning-Based Ensembling of Capsule Network with Incremental Extreme Learning Machines for Classification of COVID-19 Using CT Scans

**DOI:** 10.3390/bioengineering10020203

**Published:** 2023-02-03

**Authors:** Hassaan Malik, Tayyaba Anees, Ahmad Naeem, Rizwan Ali Naqvi, Woong-Kee Loh

**Affiliations:** 1 Department of Computer Science, University of Management and Technology, Lahore 54000, Pakistan; 2 Department of Software Engineering, University of Management and Technology, Lahore 54000, Pakistan; 3 Department of Unmanned Vehicle Engineering, Sejong University, Seoul 05006, Republic of Korea; 4 School of Computing, Gachon University, Seongnam 13120, Republic of Korea

**Keywords:** data privacy, COVID-19, blockchain, federated learning, deep learning, CT scans

## Abstract

Due to the rapid rate of SARS-CoV-2 dissemination, a conversant and effective strategy must be employed to isolate COVID-19. When it comes to determining the identity of COVID-19, one of the most significant obstacles that researchers must overcome is the rapid propagation of the virus, in addition to the dearth of trustworthy testing models. This problem continues to be the most difficult one for clinicians to deal with. The use of AI in image processing has made the formerly insurmountable challenge of finding COVID-19 situations more manageable. In the real world, there is a problem that has to be handled about the difficulties of sharing data between hospitals while still honoring the privacy concerns of the organizations. When training a global deep learning (DL) model, it is crucial to handle fundamental concerns such as user privacy and collaborative model development. For this study, a novel framework is designed that compiles information from five different databases (several hospitals) and edifies a global model using blockchain-based federated learning (FL). The data is validated through the use of blockchain technology (BCT), and FL trains the model on a global scale while maintaining the secrecy of the organizations. The proposed framework is divided into three parts. First, we provide a method of data normalization that can handle the diversity of data collected from five different sources using several computed tomography (CT) scanners. Second, to categorize COVID-19 patients, we ensemble the capsule network (CapsNet) with incremental extreme learning machines (IELMs). Thirdly, we provide a strategy for interactively training a global model using BCT and FL while maintaining anonymity. Extensive tests employing chest CT scans and a comparison of the classification performance of the proposed model to that of five DL algorithms for predicting COVID-19, while protecting the privacy of the data for a variety of users, were undertaken. Our findings indicate improved effectiveness in identifying COVID-19 patients and achieved an accuracy of 98.99%. Thus, our model provides substantial aid to medical practitioners in their diagnosis of COVID-19.

## 1. Introduction

It has been determined that the coronavirus (COVID-19) epidemic is one of the deadliest illnesses now affecting a large number of individuals and has a significant negative influence on their life [1]. COVID-19, causing severe acute respiratory syndrome (SARS), is affecting a significant portion of the world’s population [2,3]. The identification of COVID-19 remains one of our highest research priorities because it is highly contagious [4]. The most convenient form of diagnosis available right now is a nucleic acid test that involves taking swabs from the pharynx [5] and the nasopharynx [6]. However, the accuracy of the diagnostic results is compromised due to inaccuracies in the samples and a low viral load. On the other side, antigen tests may be completed in a shorter amount of time, but they are not as sensitive. Imaging techniques such as chest CT and chest X-ray (CXR) can help diagnose infections in patients, in addition to the findings of pathological investigations. To improve the accuracy of detection, a significant number of DL models have been suggested. These models examine CT and CXR images to determine the many types of infection that are present [7,8,9].

DL models are trained and enhanced by using just a limited number of easily available infected samples as their data source. However, sensitivity and accuracy are still impaired since there is an insufficient amount of training data [10]. The apparent answer to this problem is to use FL in its traditional form. FL trains a global model collaboratively over a decentralized network by combining locally learned models from a variety of sources [11,12,13]. The transmission of such personally identifiable information is not feasible since healthcare organizations do not have access to a technique that protects patients’ privacy [14,15,16]. Shokri and Shmatikov [17] established a distributed paradigm to handle this problem. Within this framework, users can exchange gradients while maintaining the data’s anonymity. Despite this, their method was susceptible to assault from even non-active adversaries [18,19]. The study [20] developed a method that safeguards users’ privacy while allowing for the secure aggregation of gradients through the utilization of the federated learning global model. To ensure the safety of the gradients, Zhang et al. [21] developed two new security methods: homomorphic encryption (HE) and threshold secret sharing (TSS). The shared approach does not provide any kind of guarantee about the credibility of its users. In other words, the lack of trust across a variety of sources continues to degrade the quality of the data, which results in poor training for the models.

The technology known as blockchain provides several different trust mechanisms [22,23] that can be used to address the problem of lack of trust in a decentralized network. The idea of privately shared data served as the foundation for the data source authentication system that was later developed. A method that ensures the authenticity of the data and is based on BCT was proposed by Qu et al. [24] for the aggregation of local DL models. These methods of data sharing increase the chance of data leakage since they neglect the confidentiality of gradients. In addition, these methods need a large number of rounds for each aggregation, which is incompatible with the decentralized network used by BCT.

This research is prompted by several significant underlying issues. COVID-19 is fast spreading, and people infected with it exhibit a broad spectrum of symptoms. Consequently, hospitals can exchange their data to aid in the accurate diagnosis of COVID-19 patients. The issue of securely exchanging data (without jeopardizing the privacy of the users) and developing a global model for the identification of positive situations is difficult. Additionally, the current study cannot jointly exchange data to successfully train the model. An important barrier that must be overcome to build AI-based methods is the collection of data from a wide variety of sources. It is not possible to make such personally identifiable information available [15,16,17,18] since there is no technique that healthcare facilities can use to protect patients’ confidentiality. A further challenge is posed by the necessity of training the DL model collaboratively across a public network.

The most recent study conducted by the World Health Organization (WHO) indicates that COVID-19 is an infectious illness that mostly affects the lungs, giving them a honeycomb-like appearance as a result. Even after making a full recovery from COVID-19, some people are nevertheless left with persistent lung impairment. The primary motivation of the present study is to locate tiny COVID-19-infected areas in the lungs. This identification has the potential to assist highly trained radiologists in more accurately locating sick regions. The second motivation for sharing data is to construct a more effective model for DL because data providers are understandably concerned about their personal information being shared. The sharing of data makes it easier to construct a viable model that is based on deep learning for automatically identifying COVID-19 patients.

To solve the challenges that have been outlined above, we present an approach that creates a precise collaborative model by utilizing data from five different databases to identify CT scans of COVID-19 patients. The blockchain-based FL architecture that has been suggested gathers data from many hospitals, each of which uses a different kind of CT scanner. Initially, we propose a process for data normalization to standardize the various sources of data. Then, to find COVID-19 patterns in lung CT scans, we make use of five different DL models, such as VGG-16, VGG-19, ResNet-101, DenseNet-169, and DenseNet-201. For CT scan image segmentation, we used a convolutional–deconvolutional capsule network, called SegCaps [25], and a CapsNet [26] with a combination of IELMs trained for improved generalization. While compared to the performance of other DL models, we found that the performance of CapsNet when using IELMs was significantly better. We eventually trained the global model using the FL technique, which allowed us to address and overcome the privacy challenge. The proposed architecture starts with a collection of data and then jointly trains an intelligent model. After that, the model is shared and spread over the public network. By utilizing FL, the weights from the many local models may be integrated without compromising the hospitals’ right to the privacy of their patient information. Only gradients are shared with the blockchain network by hospitals to maintain the secrecy of patient information. The FL system, which is powered by blockchain, compiles gradients and sends updated models to hospitals that have been verified. The decentralized design of blockchain for the exchange of data between several hospitals allows for the safe sharing of data without compromising the hospitals’ right to personal privacy.

The key contributions of the present work are given below:

1. In the study, spatial and signal normalization techniques are used for data normalization as the CT scans data are acquired from a variety of sources (i.e., hospitals and devices).

2. The suggested system can identify and classify COVID-19 patterns from CT scans obtained from a variety of sources by utilizing an ensemble of CapsNet with IELMs. The IELMs inside the capsule are used to enhance feature extraction and categorization.

3. This research describes the data collecting and sharing unit that is driven by BCT. This unit collects data from a variety of sources and utilizes FL to protect the anonymity of data shared across organizations while providing high-precision global model training.

4. The superiority of the proposed technique is proved by comparing its performance metrics to those of other existing DL algorithms making use of datasets derived from a variety of different sources.

The structure of the present study is as follows: In Section 2, relevant studies on the decentralized network are presented. Section 3 presents the methodology of the study. This section contains the CT scan image normalization techniques, dataset description, ensembling of CapsNet with IELMs, and performance evaluation metrics. Section 4 consists of results and discussions. Finally, this study is concluded in Section 5.

## 2. Literature Review

This section presents the recent literature for the classification of COVID-19 and other chest diseases using different DL, machine learning (ML), and FL. To make a prompt diagnosis of the illness, Chattu et al. [27] assessed the most recent techniques in medical imaging analytics in terms of prediction, electronic therapy, stage classifications, virtual monitoring, and data transmission. The supervised learning classifiers, such as SVM, DT, KNN, and ANN, are utilized in the field of medical imaging for the detection of chest diseases. In a similar vein, the BCT is indispensable for public access to medical data as well as worldwide data transfer that makes use of a dispersed network of many blocks. The author [27] concluded that the most recent technological developments that benefit the medical industry are the ones that control the transmission of modern medical images. In the study [28], they designed a “blockchain federated learning” architecture, which they referred to as BlockFL, for decentralized FL. The “BlockFL” design was successful in avoiding the need for a single point of failure by expanding the scope of its federated reach. The outputs of local training are included in the verification process so that devices can obtain access to public networks. To address issues regarding data privacy and security, Martinez et al. [29] make use of a cryptocurrency and FL. A systematic library of off-chain recordings is proposed by the authors as a means of scaling gradient recording and reward. He et al. [30] provided experimental research on automated FL (AutoFL), which seeks to improve the quality and productivity of local ML models that link their model updates by utilizing a neural architecture search (NAS) and a federated neural architecture search (FedNAS). They discovered that the default configurations of local ML models did not work well in a federated context for clients with non-IID (also known as non-unique ID). FLchain, as presented in [31] is a centralized, publicly verified, and healthy FL ecosystem in terms of trust and incentive. FLchain eliminates the requirement for a typical centralized FL coordinator by leveraging BCT.

When it comes to working with small datasets, Parnian et al. [32] developed a model called COVID-CAPS that is built on capsule networks as a solution to the limitations that CNN-based models present. This model has the capability of identifying COVID-19-positive cases in CXR. After making several adjustments to the parameters of the model, it was found that the COVID-CAPS model performed better than the conventional network. Studying the architecture of a blockchain network using global models is possible if one makes use of the concept of channels. In the study [33], they suggested the design of the blockchain be built on channel-specific distributed ledgers. To adhere to the decentralized data maintenance strategy, each locally relevant model parameter is saved as a block in a distributed ledger that is particular to the channel. If one applies the concept of channels, it is possible to investigate the architecture of a blockchain network using global models. Xuan et al. [34] proposed in their work that the blockchain should be constructed using channel-specific distributed ledgers as its underlying data structure. Each locally relevant model parameter is stored as a block in a distributed ledger that is specific to the channel to comply with the decentralized data maintenance strategy.

Salam et al. [35] proposed a new FL approach for patients with COVID-19. The recommended neural network, which has been pretrained using CXR photographs of abnormal patients, forecasts the severity of death and offers electronic therapy. Because of its limitations, the predictor that was developed is not suitable for use with big data sets. PriMIA is an open-source software framework for medical imaging that was developed by Kaissis et al. [36]. It is built on FL and works with many different sources of pediatric radiology with the goal of categorization. Utilizing a trained pediatric CXR image collection, DCNN which is a part of the proposed PriMIA is designed to classify the various phases of cardiac illness. A gradient-based model is used during the training of DCNN so that it may detect chest infections at an earlier stage. In a study [37], they designed an artificial intelligence (AI)-based model named EXR for the classification of COVID-19 patients using CXR images. Additionally, FL was also utilized to make the user data secure. Their proposed model achieved an area under the curve (AUC) of 0.92. Dou et al. [38] developed a novel model with the help of FL configurations and convolutional neural network (CNN) to detect lung abnormalities that occur due to COVID-19 using CT scans. They achieved a specificity of 95.27%. The availability of local annotations, as well as their level of quality, are often subject to a wide range of variation. This is mostly attributable to the fact that medical equipment and resources are distributed differently across the world. Because the major patterns differ in size, shape, and texture, this might potentially have a substantial impact on the process of identifying COVID-19. In their paper, Yang et al. [39] present a solution to this problem by making use of FL and semi-supervised learning. To study the performance gap that might occur when training a model on one dataset and then applying that model to another dataset, a multinational database was used. This database had 1704 scans from three different countries. Radiologists with years of experience manually highlighted 945 photos for the COVID-19 findings. The proposed structure produces accurate results that are also efficient to the extent of 71%. Kumar et al. [40] provide a method that trains a global DL model with blockchain-based FL using a limited amount of data that comes from a variety of sources (various hospitals). Their proposed model achieved an 83% accuracy. Podder et al. [41] proposed an LDDNet model for the classification of COVID-19 and pneumonia by using CXR and CT scan images. They used different optimizers such as Adam, Nadam, and SGD. In the end, the LDDNet model achieved significant results while using the Nadam optimizer. In the study [42], authors proposed an automated tool for COVID-19 diagnosis called RADIC. The proposed model RADIC combines time-frequency data with information collected via multiple radiomics to improve diagnostic performance. El-Bana et al. [43] proposed a multi-task model that was used for the segmentation and classification of COVID-19 from CXR and CT scan images. Shah et al. [44] proposed a CNN-based model named CTnet-10 and achieved a diagnostic accuracy of 82.1% for COVID-19 and non-COVID-19 cases using CT scans. Attallah [45] provides a deep-learning-based pipeline that is called CoviWavNet. This pipeline can automatically diagnose COVID-19. The OMNIAHCOV 3D Multiview dataset is utilized by the CoviWavNet algorithm. In the beginning, the CT slices are analyzed using multilevel discrete wavelet decomposition (DWT), and the heatmaps of the different approximation levels are used to train three ResNet CNN models. Using the spectral and temporal information included inside these photos, these ResNets can perform the categorization. After that, they investigate whether or not merging the geographical information with the spectral–temporal information could improve the diagnostic accuracy of COVID-19. Transfer learning is used to extract deep spectral–temporal characteristics from these ResNets, and then those characteristics are combined with deep spatial features that were extracted from the same ResNets that were trained on the initial CT slices. After that, they put these integrated features through a feature selection step to reduce the dimension of those features, and then they input those features into three support vector machine (SVM) classifiers. SARS-COV-2-CT-Scan, which is a benchmark dataset that is available to the public, was used to further validate the performance of CoviWavNet. According to the results of CoviWavNet, training the ResNets using the spectral–temporal information of the DWT heatmap photos is better than training them with the spatial information of the original CT scans. In the research [46], the authors describe a model for computer-assisted diagnosis that is founded on several DL and texture-based radiomics approaches. When training ResNet-18 (83.22%, 74.9%), ResNet-50 (80.94%, 78.39%), and ResNet-101 (80.54%, 77.99%), using texture-based radiomics images (gray-level covariance matrix, discrete wavelet transform) is more accurate than using the original CT scan images (70.34%, 76.51%, and 73.42%) for ResNet-18, ResNet-50, and ResNet-101, respectively. The study [47] designed a DL model for the classification of COVID-19, pneumonia, and healthy cases using CT scan images. Shankar and Perumal [48] presented a one-of-a-kind fusion model, which they referred to as the FM-HCF-DLF model. This model was designed particularly with deep learning features in mind, and it was supplied for the aim of diagnosis and categorization of COVID-19. The findings of the experiments demonstrated that the proposed model offered superior performance. It had a maximum sensitivity of 93.61%, a specificity of 94.56%, a precision of 94.85%, an accuracy of 94.08%, an F score of 93.2%, and a kappa value of 93.5%. Additionally, the model had a maximum accuracy of 94.08%. The DL method was developed by Liu et al. [49] to screen COVID-19-positive chest CT patients. The primary goal of the study was to provide reliable and timely information that could be used to enhance disease monitoring in medical imaging. The proposed model takes advantage of both spatiotemporal and dimensional properties. In addition, a segmentation model was developed in conjunction with a completely connected CRF to enhance the ROI input. With a mean dice percentage of 91.24 percent, the recommended method successfully segregated COVID-19 lesions. We summarize the recent work that uses FL and DL models for the identification of COVID-19 using CT scans and CXR in Table 1.

## 3. Materials and Methods

In this section, we discuss detailed information on the COVID-19 datasets. Afterward, the proposed methodology is presented, which is further divided into two sections: (1) local client model and (2) blockchain-based FL. The data normalization techniques have also been explained, which are used to normalize the CT scans. Additionally, the performance evaluation metrics have also been described in this section.

### 3.1. Dataset Description and Data Augmentation

AI has always occupied a key position in the field of clinical care. In environments as chaotic as these, AI can aid medical professionals in confirming the technique for sickness diagnosis. As a direct result of this, diagnostic techniques will become more accurate, which will ultimately save a great number of lives. At the moment, the most significant barrier that stands in the way of AI-based methods is the availability of relevant data. The advancement of AI is impossible without ready access to vast amounts of pertinent data. Diverse CT images with varied characteristics were gathered from data sources utilized to train the suggested model. Datasets 1, 2, 3, 4, and 5 are used to describe the COVID-19 databases used for assessing the model.

#### 3.1.1. Dataset-1

Patients with COVID-19 infections that have been verified and who have had unenhanced chest CTs make up the first dataset [56]. Patients most frequently described having hypertension, diabetes, and pneumonia or emphysema as co-occurring conditions in their medical histories. Between March 2020 and January 2021, patients who had a positive RT-PCR test result for COVID-19 and accompanying clinical symptoms were photographed within an inpatient setting. During the CT exams, which were carried out on a NeuViz 16-slice CT scanner and conducted in the “Helical” mode, no intravenous contrast was administered. A dataset contains a total of 35,635 CT scan images including 9367 CT scans of normal cases. Every image is in the DICOM format and is formed of grayscale depictions that are 512 pixels wide and 512 pixels high.

#### 3.1.2. Dataset-2

The second dataset, known as the CC-19 [57] dataset, has a total of 34,006 CT scan slices, which were contributed by 89 individuals from three hospitals. Of them, 28,395 CT scan slices belonged to patients who tested positive for COVID-19. The data, which consists of CT scan slices for 89 different people, have been scanned by three different scanners (such as Brilliance ICT, Samatom definition Edge, and Brilliance 16P CT) that are independent of one another. There were 89 participants examined, and 68 of those subjects tested positive for the COVID-19 virus. The remaining 21 persons tested negative for COVID-19.

#### 3.1.3. Dataset-3

The third SARS-CoV-2 CT scans dataset was collected from [57,58]. The dataset has a total of 2482 CT scans, 1252 of which are positive and 1230 of which are from patients who are not infected with SARS-CoV-2 [59]. The information was gathered from actual patients who were treated at hospitals located in Sao Paulo, Brazil.

#### 3.1.4. Dataset-4

The fourth database, Mosmed-1110 [60], contains 1110 three-dimensional CT scans of patients that were performed on them while they were being treated in hospitals in Moscow, Russia. There are five distinct kinds of 3D CT volumes included in this dataset. The labels CT0, CT1, CT2, CT3, and CT4 were given to these groups. The CT0 consists of 254 volumes of 3D CT, all of which are considered to be normal scans. If the person’s level is normal, it means that the CT scan did not reveal any symptoms of pneumonia or COVID-19. CT1 is comprised of 684 different 3D CT scans, and these scans reveal that 25% of the patient’s lungs are infected with COVID-19. CT2 consists of 125 different 3D CT scans, and the results of these scans suggest that the patient’s lungs were infected with COVID-19 between 25% and 50% of the time. In addition, CT3 consists of 45 3D CT scans, and these scans reveal that the patient’s lungs were infected with COVID-19 between 50% and 75% of the time. The CT4 category requires two 3D CT scans to be performed on lungs that are 75% to 100% infected with COVID-19. In addition, levels of mild (CT1) or moderate (CT2) indicate that the patient does not require any intensive care in a hospital and may, as a result, continue to live at home. In contrast, patients in the severe (CT3) and (CT4) critical phases are required to remain in the hospital throughout their treatment under intensive care. For this study, we only considered two categories CT0 and CT2 for classifying normal and COVID-19-infected individuals.

#### 3.1.5. Dataset-5

In dataset 5 [60], there are a total of 349 COVID-19 CT scans taken from 216 individuals in addition to 463 non-COVID-19 CT photos. During an epidemic, patients whose presence of SARS-CoV-2 was verified by RT-PCR were recruited at the point of care to contribute to this dataset.

**Figure 1:**
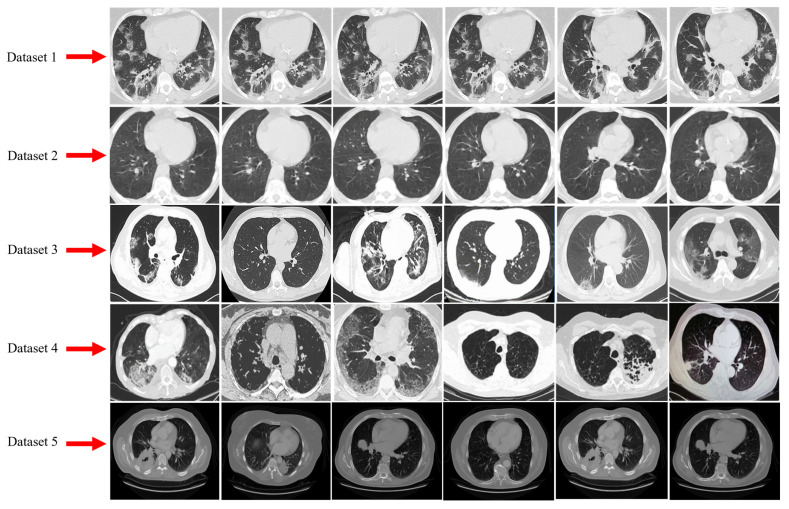
Samples of CT scan images of COVID-19 were collected from Datasets 1 to 5.

Table 2 presents the complete statistics of all five datasets and a few samples of CT scans of COVID-19 infected collected from these datasets are shown in Figure 1. Additionally, it has been observed that the number of images in each dataset is imbalanced. Therefore, the synthetic minority oversampling technique (SMOTE) [61] is utilized to balance the datasets.

### 3.2. Overview of FL and Blockchain

**Figure 2:**
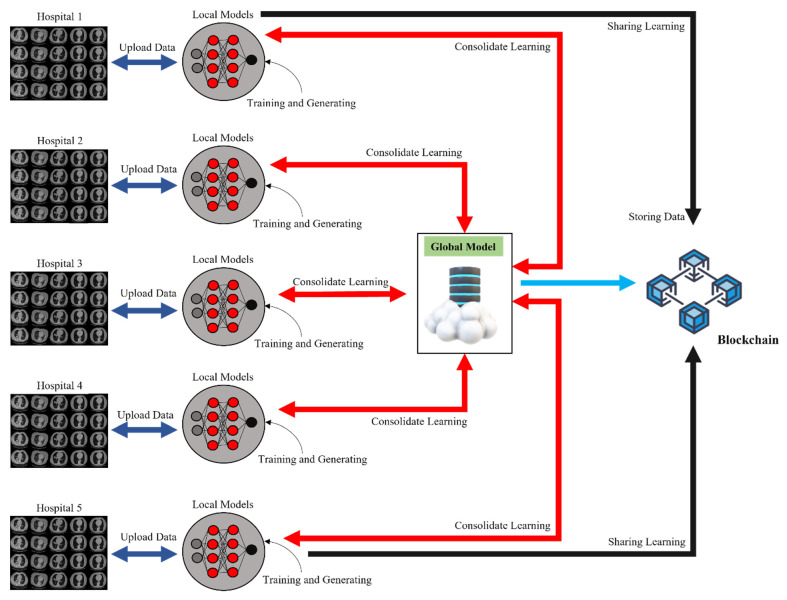
Proposed blockchain-based FL framework used for the identification of COVID-19 through CT scans.

For the present work, we disseminated the data to five hospitals. The data from each hospital is added to the global model as shown in Figure 2. The major purpose of this study is to facilitate the sharing of data from a wide variety of sources and the collaborative training of a DL model. We create a normalization technique to handle the many different kinds of CT scanner data as a result of the fact that the data is obtained from a variety of sources. Following the completion of the data normalization process, the CT scans were segmented, and then a model for the classification of COVID-19 suspects was trained using CapsNet in conjunction with IELMs. We make use of an FL architecture that is built on BCT to train and disseminate a collaborative model. The weights of locally trained models are intended to be included in FL’s design. When all of the weights from the locally trained models have been combined, the global model is brought back to the hospitals.

The right of the data providers to maintain their privacy is the factor that has the highest weight. The blockchain is used to regulate both the privacy of sensitive data and its exposure. As a result, we make entries in the blockchain ledger for the two distinct types of transactions: (A) transactions involving data sharing and (B) transactions involving data retrieval. To control data accessibility while maintaining data privacy, this research makes use of a blockchain that requires permission to access. A permissioned blockchain’s ability to track all transactions and provide access to data derived from a global model is the fundamental advantage of using a blockchain. The second objective is to organize efforts on the data. The blockchain FL solution that has been proposed combines the weights of the local models and then updates its weights. We develop a local model for the data that is diverse or imbalanced by using a process called spatial normalization.

The model that has been proposed is made up of two different parts. (1) the local client model and (2) the FL framework based on BCT. We start by finding a solution to the problem of the CT scan data being of varying types. Then, to segment the data, we make use of SegCaps [25] and train a local model to identify COVID-19 pattern sequences by using an ensemble of CapsNet with IELMs. In the last step, the blockchain network is provided with the weights of the local models so that they may be used to train the global model.

### 3.3. Local Client Model

In this section, we discuss the normalization approaches, segmentation of the data, and the DL model i.e., CapsNet with IELMs.

#### 3.3.1. Data Normalization

The fact that FL must deal with input data coming from a variety of sources and machines that each have their unique parameters is a significant challenge. The majority of the currently available approaches are wholly inadequate to solve this problem for FL. To overcome this impediment, we have devised a method of normalization that applies to a wide variety of CT scan modalities and aligns the images to the same level of quality. By performing this phase of normalization, FL can deal with the unpredictability of the dataset and construct a more efficient learning model. The process of normalizing the COVID-19 CT scans is divided into two stages: the spatial normalization stage, and the signal normalization stage. The size and resolution of the CT scan are taken into consideration by spatial normalization. The term “signal normalization” refers to the process by which CT scanners adjust the intensity of each voxel based on the lung window.

- Spatial Normalization of COVID-19 CT scans

The sizes and resolutions of CT scan images serve as the primary determinants of the spatial normalization of the images. As discussed in the studies [40,61], we used a standardized volume of 332 × 332 × 512 mm^3^ for human lung CT scans. This technique of normalization is used for all of the obtained datasets, and all CT scan image formats are transformed into a common format [62] to make FL possible. As a consequence, both learning and performance are enhanced.

- Signal Normalization of COVID-19 CT scans

During signal normalization, the intensity of each voxel is determined by computing it relative to the lung window. Window level (W_level_) and window width (W_width_) are two common types of windows that are used in medical procedures. This is because every CT scan requires Hounsfield units (HU). Using Equation (1), we can obtain the normalized value using this window size as the parameter.

(1) $$C_{normalized}=\frac{C_{original}-W_{level}}{W_{width}}\;$$

The original CT scan image is denoted by *C_original_*, while the image that has to have its intensity normalized is denoted by *C_normalized_*. In this particular experiment, we select the lower-limit window size ranges between −0.05 and 0.5.

#### 3.3.2. CT Scans Based Segmentation and Classification for COVID-19

As discussed in [25], we used SegCaps to perform the segmentation process of COVID-19-infected CT scan images. In addition, segmented CT scan images are used throughout the training process for COVID-19 recognition by the CapsNet in combination with IELMs. When it comes to lung segmentation, the suggested approach uses 2D slices as the input. For the segmentation of human lungs, a volume with the dimensions 334 × 334 × 512 mm^3^ is employed. Every three-dimensional (3D) CT scan volume consists of three planes: CX, CY, and CZ. We define these planes so that lung infection may be distinguished more readily. In addition, Equation (2) is used to measure these planes of 3D CT scans.

(2) $$P^{I}=D\;\left(R_{CX}^{I},\;R_{CY}^{I},\;R_{CZ}^{I}\right)$$

where *P^I^* stands for the probability of infection and I refer to the site of the infection. The method that is used to define voxel views in 3D is denoted by the letter D. The *R_CX_*, *R_CY_*, and *R_CZ_* voxels may all be predicted with the use of the function I. Because the traditional approach requires a lot of time, we have modified Equation (2) to read as follows:

(3) $${\hat{P}}^{I}=D\;\left({\hat{R}}_{CX}^{I},\;{\hat{R}}_{CY}^{I},\;\;{\hat{R}}_{CZ}^{I}\right)$$

(4) $${\hat{P}}^{I}=D\;\left(f_{CX}^{I}\;\left(R_{CX}^{I}\right),\;f_{CY}^{I}\;\left(R_{CY}^{I}\right),\;f_{CZ}^{I}\;\left(R_{CZ}^{I}\right)\right)$$

#### 3.3.3. Ensembling of CapsNet with IELMs

In recent years, the DL framework has seen an increase in popularity because of the powerful feature extraction layers and classification procedures that it provides. In recent years, CNN has seen an increased amount of use for CT scans image classification analysis [56,57,58,59,60,61]. This leads to a large increase in the amount of computing complexity and even affects the level of performance achieved by the classifier. This is because the pooling layers of a CNN do not examine the spatial link between the features in an image [59]. The method that was suggested combines the potent properties of CapsNet with IELMs to get around the issues that were discussed earlier to achieve improvements in classification accuracy and diagnosis. CapsNet is used in this technique to produce robust feature maps, and IELMs have been used as substituted for conventional dense classification layers to improve COVID-19 prediction.

1. CapsNet Architecture

CapsNet, in contrast to Artificial Neural Networks (ANNs) [60], conducts computations on its inputs before encapsulating the findings in a compact vector of highly informative outputs. A CapsNet might be considered an alternative to ANNs in certain contexts. A comprehensive analysis between CapsNet and the traditional ANN is shown in Table 3.

**Figure 3:**
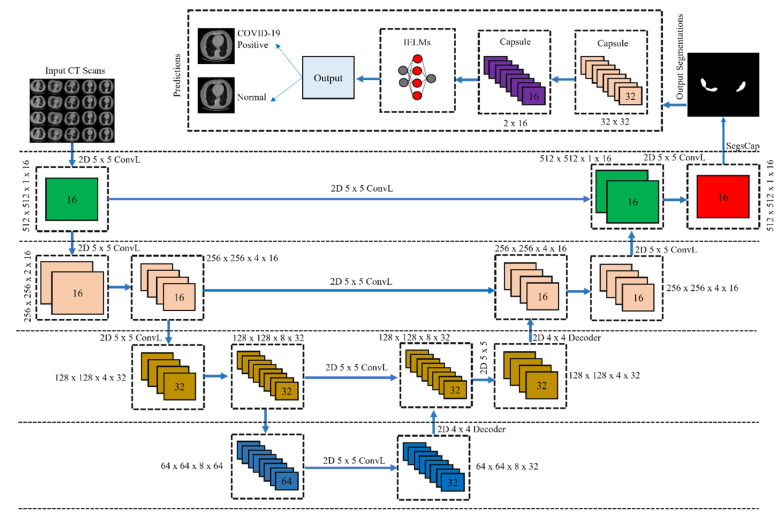
Proposed CapsNet with IELMs architecture.

The architecture of CapsNet includes a separate encoder and decoder for each of the network’s tiers. The convolutional layer (ConvL), PrimaryCaps layer (PCL), and DigitCaps layer (DCL) make up the encoder, whereas the decoder is made up of three fully-connected layers (FCL) [60]. Figure 3 presents the proposed CapsNet with IELMs used for the classification of COVID-19. The initial ConvL of the proposed CapsNet is made up of 512 kernels that are 5 *×* 5 *×* 1 in size and have a ReLU activation function. Additionally, the value of stride is set to 1. This layer is in charge of converting the intensity values of individual pixels into the activity levels of nearby feature detectors. The results of this translation are then sent to the PCL. A convolutional layer is known as PCL, which has 32 channels of 8D convolutional capsules (each capsule contains 8 convolutional units with a 5 *×* 5 kernel and a stride of 2. PCL is responsible for the generation of original CT scan images, for which it does inverse graphics [63,64,65,66,67,68] and reverse engineering [69,70,71]. A 6 *×* 6 eight-dimensional output tensor is produced as a consequence of the capsule’s application of eight 5 *×* 5 256 kernels to an input volume of 20 *×* 20 *×* 256. The output would have the dimensions 6 *×* 6 *×* 8 *×* 32 given that there are 8D capsules in the system. Each of the 16D capsules that make up a class in the DCL receives its input from the capsule that comes before it in the stack. To perform an affine transformation on each 8D capsule, the 8 *×* 16 weight matrix (M_(a,b)_) is used. Additionally, Equation (5) was applied to encode (E_(a,b)_) the critical spatial link that exists between low-and high-level features within the CT scan image.

(5) $$E_{a,b}=M_{\left(a,b\right)}*\;V_{\left(a,b\right)}*\;I_{\left(b\right)}$$

whereas I_b_ is the input vector, M_(a,b)_ shows the weight matrix, and V_(a,b)_ is the component vector.

To obtain the current capsule “C”, we use Equation (6) to find the sum of the input vectors with their respective weights.

(6) $$G_{k}=\sum_{b}E_{\left(a,b\right)}*\;C_{\left(p,q\right)}$$

Equation (7) is derived by putting the value of Equation (5) into Equation (6) which is used to measure the weighted sum of the input vector.

(7) $$G_{k}=\sum_{b}(M_{\left(a,b\right)}*\;V_{\left(a,b\right)}*\;I_{\left(b\right)})*\;C_{\left(p,q\right)}$$

where *C_(p,q)_* can be represented as a routing softmax function (RSF). Equation (8) is used to measure the RSF.

(8) $$C_{\left(p,q\right)}=\frac{e^{b_{\left(p,q\right)}}}{\sum_{L}e^{b_{\left(p,L\right)}}}\;$$

Equation (7) is further modified by adding the value of *C_(p,q)_* from Equation (8). The resultant value for measuring the weighted sum of the input vector is shown in Equation (9).

(9) $$G_{k}=\sum_{b}(M_{\left(a,b\right)}*\;V_{\left(a,b\right)}*\;I_{\left(b\right)})*\left(\frac{e^{b_{\left(p,q\right)}}}{\sum_{L}e^{b_{\left(p,L\right)}}}\right)$$

Scaling the probability from 0 to 1 is accomplished by using a squashing function, which is computed by Equation (10).

(10) $$S_{K}=\frac{||S_{K}|{|}^{2}}{1+||S_{K}|{|}^{2}}\;\frac{S_{K}}{\left||S_{K}|\right|}$$

The ratio of “low-level capsule to high-level capsule” is altered incrementally in response to the results, to achieve an appropriate ratio. By employing Algorithm 1, we can implement agreement-based routing of the CapsNet model. The goal of a routing algorithm is to go through several iterations, during which the distribution of the output of the low-level capsule to the output of the high-level capsule is gradually modified based on the output of the high-level capsule, and an optimal distribution is eventually achieved.
**Algorithm 1: Routing Algorithm for Processing all Capsule****Input Parameters:** Capsule = C; Layers = L; Weighted Sum = W_s_**Output:** Distributing the output of low-level to high-level capsule**1.** Foreach C in L:**2.**  C (L + 1) do W_s_ = 0**3. While** K = 1:**4.** C in L: **5.  do** C_(p,q)_    // see Equation (8)**6.** Foreach C in L + 1: **7.  do** G_K_, S_K_      // see Equation (7)**8.** Foreach C of j in L + 1:**9.  do** E_(a,b)_     // see Equation (5)**10.** Foreach C of K in L AND j in L + 1 **11.  do** b_(p,q)_ ← b_(p,q)_ + $\hat{A}j$|k **12.** Return b_(p,q)_

2. IELMs

The proposed research makes use of CapsNet for enhanced feature extraction; this might be of assistance in reaching the highest possible classification accuracy. In addition, these features are then sent through to the IELMs, who are the ones responsible for classifying the CT scan images. IELM is a kind of neural network with just one hidden layer and operation based on the auto-tuning feature seen in Figure 3. The neural network utilizes a single hidden layer, which does not need any adjustments to be made to it. IELM makes use of the kernel function to obtain high accuracy to improve its overall performance. Because IELMs use autotuning of weight biases in conjunction with non-zero activation functions, the key advantages it offers are enhanced approximation and reduced training error. The studies [72,73] provide an in-depth analysis of the IELM’s operating mechanism.

The following Equations (11) and (12) are used to measure the output of IELMs:

(11) $$F_{E}=\sum_{i=1}^{E}B_{i}*M_{i}\left(X\right)$$

(12) $$F_{E}=\sum_{i=1}^{E}B_{i}*M\left(W_{i}*\;X_{K}+V_{i}\right),\;K=1,2,3,4,\ldots ,\;N.$$

Whereas the variable *X* is used for the input vector, *V* represents the vias vector, *N* represents the total number of training samples, the value of *K* shows the weight vector between the hidden and output layer, *E* and *M* represent the number of hidden units and activation functions, respectively. Additionally, *W* is the weight vector between the input and hidden layer. It is quite comparable to regular ANN with backpropagation, but if you look carefully, you will notice that the weight between the hidden layer and output is referred to as Beta. For this study, this Beta matrix is peculiar because it is used as a pseudo-inverse. Thus, the above Equation (12) can be written as:

(13) G=H∗B

The Equations (14) and (15) are used to measure the value of *G*, *H*, and *B*.

(14) $$H={\left[\begin{array}{ccc} M\left(W_{1}*\;X_{1}+V_{1}\right) & \cdots & g\;\left(W_{E}*\;X_{1}+V_{E}\right)\\ \vdots & \ddots & \vdots \\ M\left(W_{i}*\;X_{N}+V_{N}\right) & \cdots & g\;\left(W_{E}*\;X_{N}+V_{E}\right) \end{array}\right]}_{N*E}$$

(15) $$B={\left[\begin{array}{c} B_{1}^{T}\\ \vdots \\ B_{E}^{T} \end{array}\right]}_{E*M}\;,\;G={\left[\begin{array}{c} G_{1}^{T}\\ \vdots \\ G_{N}^{T} \end{array}\right]}_{N*M}\;$$

where *H* represents the hidden layer output matrix. *M* and *G* represent the number of outputs and training data target matrix, respectively. The infectious impact of COVID-19 on the lungs has been well characterized by using Equations (11)–(15). Algorithm 2 provides a step-by-step explanation of the proposed CapsNet with the IELM model.
**Algorithm 2: Pseudo Code of Proposed CapsNet with IELMs****Input Parameters:** Input Image = I_input_; No. of iteration = N_iterations_; Capsule = C; Image Features = F_image_; Threshold = T_threshold_; IELMs = E_IELM_; Output Image = O_output_; Disease = D.**Output:** Classification of COVID-19-infected CT scans**1. For** n =1:**2.**  F_image_ = C (I_input_)**3.** Execute Algorithm 1**4.** O_output_ = E_IELM_ (F_image_) // see Equation (12)**5. If** O_output_ == T_threshold_**6.**  D is **True**  // COVID-19 detected**7. Else****8.**  D is **False**  // Normal**9.** End

### 3.4. DL Classifiers

In this study, the classification accuracy of the proposed CapsNet with IELMs model was compared with five different DL methods such as VGG-16 and VGG-19 [74,75], ResNet-101 [73], DenseNet-169, and DenseNet-201 [75]. All these models are pre-trained with ImageNet [76]. The VGG network is constructed with the help of very small ConvL filters. The VGG-16 [73] consists of 13 layers of convolutional processing and three levels of FCL. The VGG19 model is identical to the VGG16 model with the exception that it supports 19 layers. The “16” and “19” represent the model’s number of weight layers. This indicates that VGG-19 has three more ConvL, compared to VGG-16. ResNet architecture is a kind of ANN that allows layers to be skipped without negatively impacting performance [73]. Additionally, ResNet-101 [77] has a total of 101 layers of depth. Conv, MaxPooling layers, dense layers, transition layers, and dense layers are the components that make up the architecture of DenseNet-169 and DenseNet-201, respectively. The ReLU activation function [66,67,68] and the SoftMax activation function [69,70,71] are both included in the overall design of the DenseNet-169 and DenseNet-201 models.

### 3.5. Blockchain-Based FL

In this part of the article, we take a look at a proposed situation involving the decentralized exchange of data across five separate hospitals. Each hospital agreed to contribute the locally trained model (weights) that it has developed, and the method that we have proposed helps hide user data while also sharing the model over a decentralized network. In addition to this, FL is used in the process of aggregating the net impacts of models that are shared throughout five hospitals. Utilizing FL to exchange data across hospitals in a way that does not compromise patient confidentiality is the major goal of this work.

**Figure 4:**
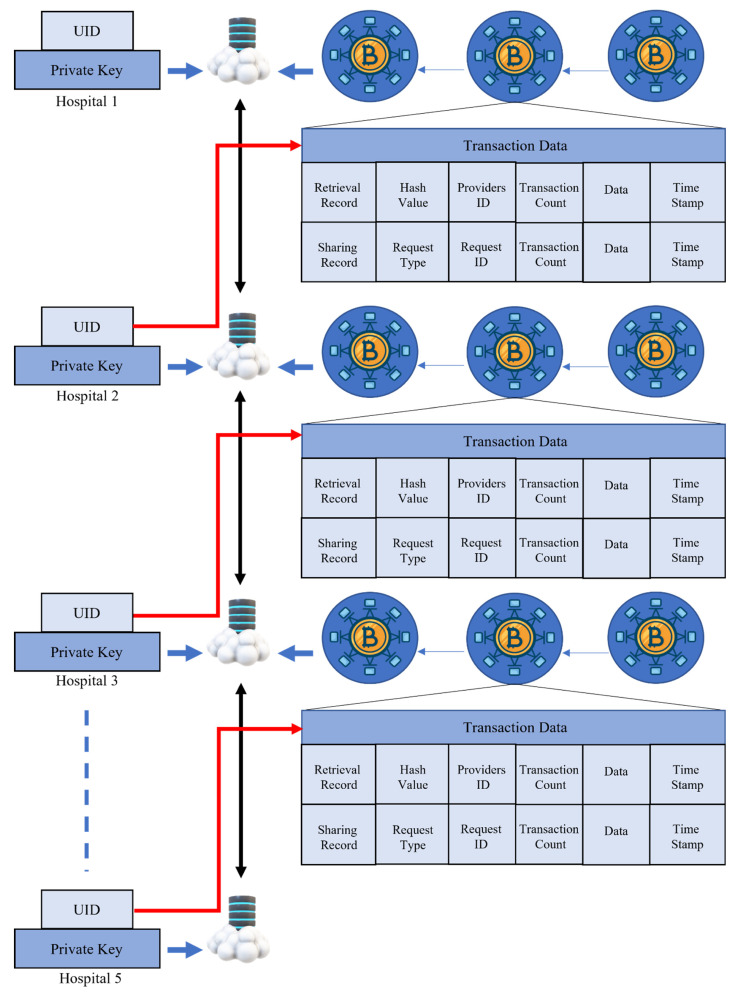
Procedure of keeping medical records on blockchain across five distinct hospitals.

It is necessary to encrypt patient data since these records are both private and take up a substantial amount of space on discs. The placing of data on the blockchain, which only has a certain amount of space available for it to store information, is expensive in terms of both cost and the resources needed to process it. Therefore, the hospital is the repository for the actual CT scan data, and blockchain serves to make it easier to obtain the trained model. When a new hospital contributes data, the block receives an update in the form of a transaction to verify that the contributor is the rightful owner of the data. The information about the hospital constitutes both the data type and the data size. Each transaction involved in the process of exchanging and retrieving data is shown in Figure 4. The proposed paradigm takes into account requirements for the retrieval of shared data. Multiple hospitals may share data and train a collaborative model to improve their ability to predict the most favorable results possible via combined efforts. The retrieval technique does not in any way violate the confidentiality of hospitals. We provide a multi-organization architecture that makes use of BCT, which was influenced by the work presented in the study [78]. The data for various categories are shared throughout all of the hospitals (H). Each category of H has a distinct community, and these communities are responsible for maintaining the table log(n). The blockchain has a record of the one-of-a-kind IDs for each hospital.

Equation (16) describes the process of retrieving data into the physically existent nodes. Equation (16) is also used to determine how far apart two nodes are located, with P standing for the data categories used to collect the information from the hospitals. In addition, we calculate the distance D_i_ (P_i_, P_j_) between two nodes which are used to retrieve the information. The characteristics of the weight matrix for the node P_i_ and P_j_ are denoted by $X_{ab}^{P_{i}}$ and $X_{ab}^{P_{j}}$, respectively. The logic and the distance between the nodes are used by each hospital to establish its unique identifier (UID).

(16) $$D_{i}\left(P_{i},P_{j}\right)=\frac{\sum_{a,b\;\in \;\left\{P_{i}\cup P_{j}-{\;P}_{i}\cap P_{j}\right\}}\left(X_{\mathrm{ab}}^{P_{i}}+{\;X}_{\mathrm{ab}}^{P_{j}}\right)\;}{\sum_{a,b\;\in {\;P}_{i}\cup P_{j}}\left(X_{\mathrm{ab}}^{P_{i}}+{\;X}_{\mathrm{ab}}^{P_{j}}\right)}*log\left(D_{p}\left(P_{i},P_{j}\right)\right)$$

Equation (17) is used to measure the nodes *(P_i_, P_j_)* with UID:

(17) $$D_{i}\left(P_{i},P_{j}\right)=P_{i}\left(UID\right)⊕\;P_{j}\left(UID\right)$$

To ensure that patient information is kept private while maintaining data decentralization, the randomization method for two hospital nodes is described in Equation (18).

(18) $$P_{n}\left[V\left(N\right)\in Z\right]\leq \exp\left(\in \right)*\;P_{n}\left[V\left(N^{\prime }\right)\in O\right]$$

where $\left[V\left(N\right)\in Z\right]$ is used to present the privacy of data, N and N′ represents the data neighboring, and the outcome of the data is represented by O. Laplace (£), on the other hand, is used for the local model training (T_i_) to protect patients’ privacy across several hospitals. In our case, we are sharing the data with five different hospitals. Thus, Equations (19)–(21) are utilized to keep the data private.

(19) $${\hat{T}}_{i}=T_{i}+£\;\left(s/\in \right)$$

(20) $$s=max_{P,\;P^{\prime }}\;\left|\left|f\left(P\right),\;f\left(P^{\prime }\right)\right|\right|$$

Equation (21) is obtained after putting the value of s in Equation (19).

(21) $${\hat{T}}_{i}=T_{i}+£\;\left(s=max_{P,\;P^{\prime }}\;\left|\left|f\left(P\right),\;f\left(P^{\prime }\right)\right|\right|/\;\in \right)$$

The global model is trained using the consensus algorithm, which is carried out with the help of the local models. Since all of the nodes work together to train the model, we provide proof of work so that the data may be distributed across the nodes. The mean absolute error (MAE) is used as the accuracy measure during the training phase of the consensus method, which analyses the quality of the local models. Equations (22)–(23) describe the consensus algorithm process used by the hospitals.

(22) $$MAE\;\left(T_{i}\right)=\frac{1}{n}\sum_{i=1}^{n}\left|A_{i}-f\left(x_{i}\right)\right|$$

(23) $$MAE\;\left(P_{i}\right)=\frac{1}{n}\sum_{i=1}^{n}MAE\;\left(T_{i}\right)+G*\;MAE\;\left(T_{i}\right)\;$$

The value of *MAE (T_i_)* from Equation (22) represents that the model is locally trained. The G value from Equation (23) demonstrates the global model weight. Additionally, *f(x_i_)* shows the model prediction, while the original data is represented by T_i_ and A_i_. All of the information held by hospitals is signed and encrypted using both public and private keys to maintain patient confidentiality. As a result, *T_i_* is responsible for computing all transactions and broadcasting each model transaction. If permission is obtained, each transaction will be added to the distributed ledger and recorded there. The following steps are performed during the training of the consensus algorithm.

1. The local model T_i_ transaction is handed over from the P_i_ node to the P_j_ node.
2. Local model T_i_ is sent up to the leader via node P_j_.
3. The leader sends out a broadcast to the P_i_ and P_j_ with the block node.
4. Check that the P_i_ and P_j_ are correct, then wait for authorization.
5. Finally, the blocks should be saved in the database of the retrieval blockchain.

### 3.6. Performance Evaluation

Different evaluation metrics such as accuracy (ACU), recall (REC), specificity (SPF), F1-score, dice similarity coefficient (DSC), and precision (PRE), are used to measure the classification performance of the proposed model. Additionally, these metrics are also utilized to calculate the segmentation performance of the SegsCap model. The receiver operating characteristic (ROC) and the area under (AU) the ROC curve of the proposed model and five DL models are also measured. The relationship between REC [79] and SPF [80] is inverse: as REC grows, SPF tends to decrease, and vice versa. High REC tests will provide positive results for people with an illness, whereas high SPF testing will indicate that patients with negative results do not have a condition [81]. The following Equations (24)–(31) are used to measure these metrics.

(24) $$ACU=\frac{TP+TN}{TP+TN+FP+FN}$$

(25) $$REC=\frac{TP}{TP+FN}$$

(26) $$SPF=\frac{TN}{TN+FP}$$

(27) $$PRE=\frac{TP}{TP+FP}$$

(28) $$F1-score=2*\left(\frac{PRE*REC}{PRE+REC}\right)$$

(29) $$DSC\;\left(A,B\right)=\frac{2\left(A\cap B\right)}{A+B}$$

(30) $$TPR=\frac{TP}{TP+FN}$$

(31) $$FPR=\frac{FP}{TN+FP}$$

where *A* and *B* represent the target regions, the spatial overlap between two segmentations is measured using *DSC*, *TP* and *TN* represent the true positive and true negative, separately. *TPR* is used to represent the true positive rate, *FPR* shows the false positive rate, and *FP* and *FN* denote false positive and false negative, respectively.

## 4. Results and Discussions

In this section, we present comprehensive results obtained by using the proposed blockchain-based FL model and five different DL models.

### 4.1. Experimental Setup

The proposed model was built using open-source TensorFlow (TF) federation version (v) 2.1.0, whilst the five DL models are implemented with TF v1.8. The Keras library was also utilized as the backend for their respective implementations. In addition, Python is used in the development of processes that are unconnected to convolutional networks. The DL models were made more efficient by using an Adams optimizer to make adjustments. We also fine-tuned the DL models by using a learning rate (LR) of 0.0001, batch size of 32, a momentum of 0.9, dropout of 0.2, ReLU activation function, and 100 epochs. The experiment was carried out using a workstation that ran the Windows operating system and had 32 GB of RAM together with an 11 GB NVIDIA GPU. We split the five CT scan images dataset into 70% for training, 20% for validation, and 10% of the data used for testing. A total of 70% of the five datasets are used to train the CapsNet with the IELMs model using TF federated v 2.1.0. This same ratio of the training set is also used to train the five DL models. On the FL blockchain, these models are implemented using Python 3.9.5.

### 4.2. Comparison of the Proposed Model with DL Models

**Figure 5:**
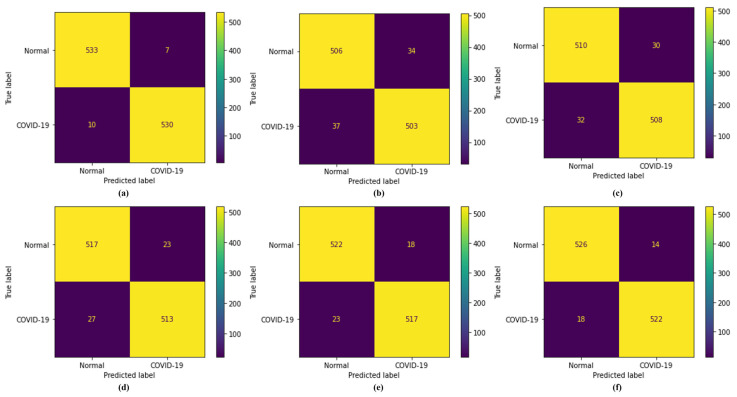
Confusion matrices; (**a**) proposed model, (**b**) VGG-16, (**c**) VGG-19, (**d**) ResNet-101, (**e**) DenseNet-169, and (**f**) DenseNet-201.

To test the efficacy of the proposed model and five DL models, many different performance metrics were examined for the accurate diagnosis of COVID-19 using CT scan images. The confusion matrices for the proposed CapsNet with the IELMs model with different DL models are shown in Figure 5. In the test set, there were 540 COVID-19 images and 540 normal CT scan images. In the confusion matrices, actual occurrences were grouped along rows, and predicted cases along columns. Among 540 COVID-19 instances, the proposed model presented classified 530 cases and incorrectly categorized 10 cases as normal. The VGG-16 model correctly classified the 503 COVID-19 cases and misclassified 33 cases as normal. Additionally, VGG-19, ResNet-101, DenseNet-169, and DenseNet-201 correctly classify the 508, 513, 517, and 522 COVID-19 cases, respectively. The detailed results are presented in Figure 5.

We carried out a comprehensive set of tests using the proposed model with five different DL models, including (VGG-16, VGG-19, ResNet-101, DenseNet-169, and DenseNet-201). DL models consisting of multiple layers were used for the analysis of the COVID-19 CT scan images dataset, and the results of these evaluations are shown in Table 4.

**Figure 6:**
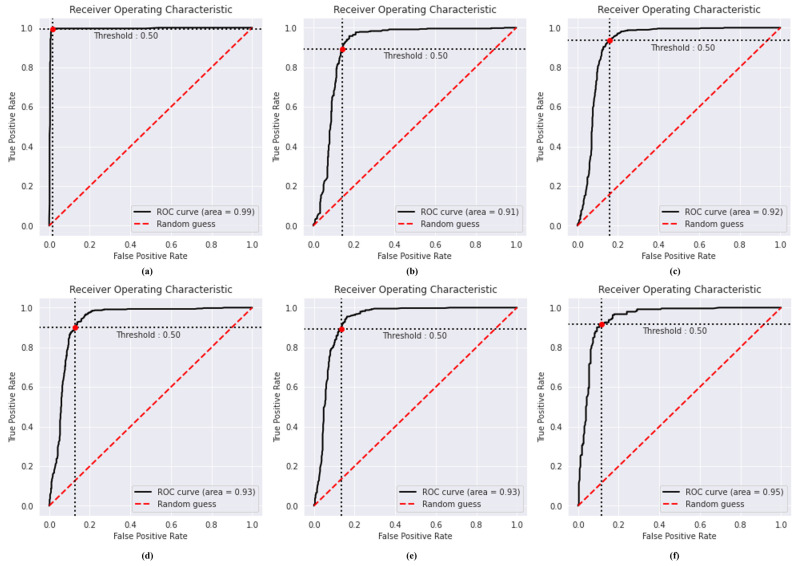
ROC curves; (**a**) proposed model, (**b**) VGG-16, (**c**) VGG-19, (**d**) ResNet-101, (**e**) DenseNet-169, and (**f**) DenseNet-201.

From Table 4, it has been observed that the proposed CapsNet with IELMs achieves the highest accuracy of 98.99%, PRE of 98.96%, REC of 98.97%, 98.95% of SPF, and 98.96% F1-score. The highest value of REC (98.97%) of the proposed model among five different DL models indicates that the model is more effective and accurate in classifying COVID-19-infected CT scans. However, the significant value of SPF (98.95%) shows that the proposed model is also effective in classifying normal cases using CT scan images. The second-highest results were obtained by the DenseNet-201. The DenseNet-201 model attains the ACU of 95.45%, PRE of 95.54%, REC of 95.47%, and F1-score of 95.48%. The VGG-16 model shows poor results in classifying COVID-19 cases using CT scan images. Figure 6 presents the performance of the proposed model in comparison to the five alternative DL models at each level of classification using a ROC curve. The two parameters that are discussed in the Equations (30) and (31) are shown on the ROC curve.

**Figure 7:**
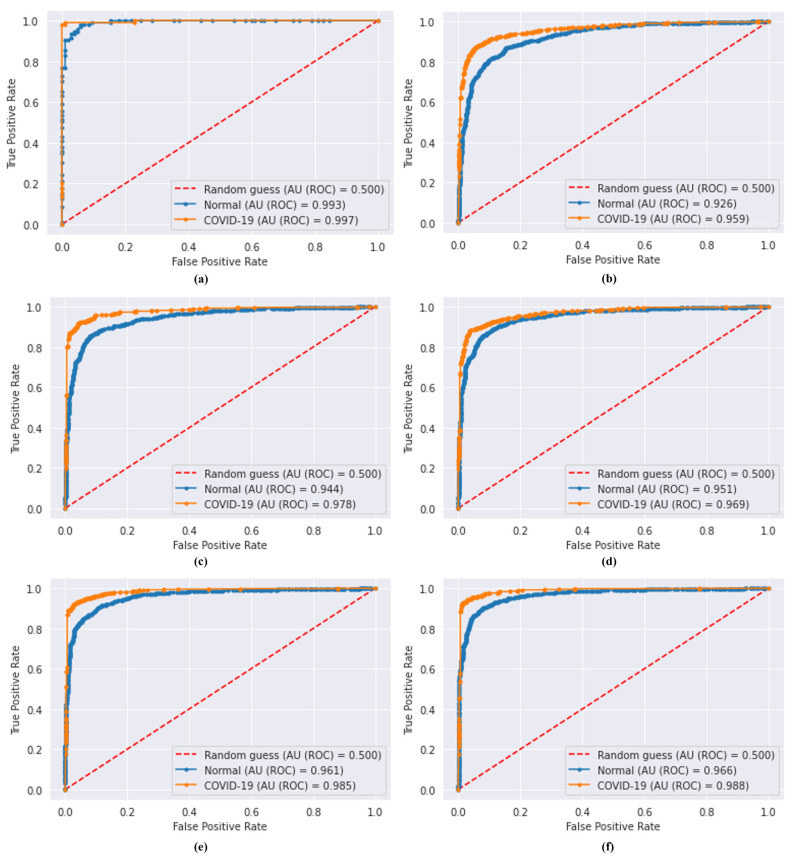
AU(ROC) curves; (**a**) proposed model, (**b**) VGG-16, (**c**) VGG-19, (**d**) ResNet-101, (**e**) DenseNet-169, and (**f**) DenseNet-201.

The higher the AU(ROC) is, the greater the model is considered to be efficient for medical diagnosis. The AU(ROC) curves of our classifiers are plotted in Figure 7. For the proposed model, the AUC was 0.997 and 0.993 for COVID-19 and normal class, respectively. For VGG-16 and VGG-19, the AUC was 0.959 and 0.978 for the COVID-19 class, respectively. For ResNet-101, the AUC was 0.969. Finally, the DenseNet-169 and DenseNet-201 achieved an AUC of 0.985 and 0.988 for the COVID-19 class, respectively. The AUC results reveal that the proposed model could contribute efficiently to classifying COVID-19 cases from CT scan images.

**Figure 8:**
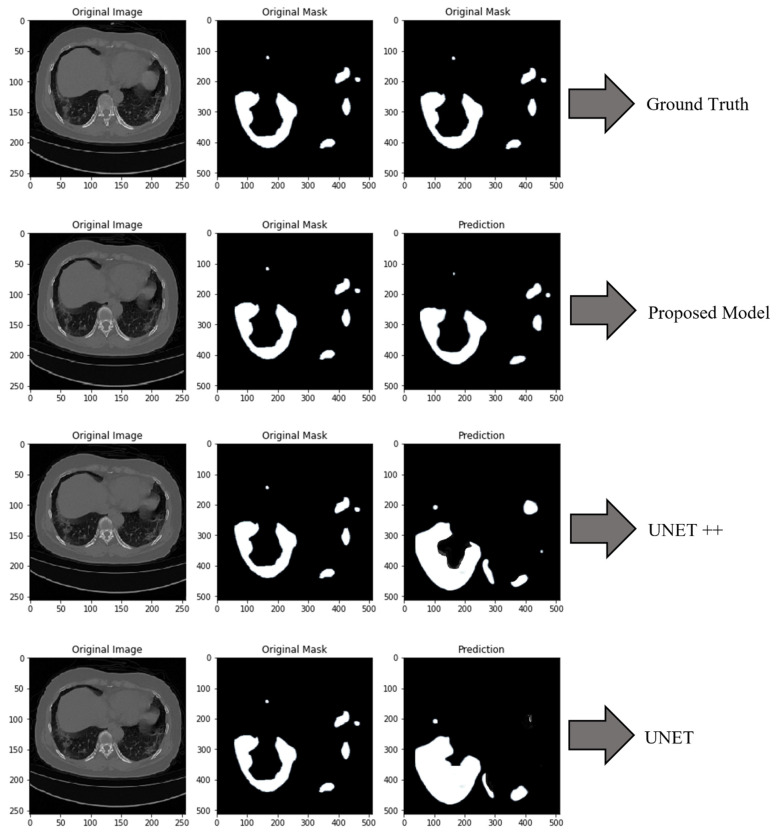
Segmentation of COVID-19 infected CT scan. The first row represents the ground truth portion of the COVID-19-infected patients. The second row presents the prediction made by the proposed model about the infected area. In the third and fourth row are presented the results produced by the UNET++ and UNET, respectively.

A total of 540 CT scan slices from each hospital were used in the process of evaluating the proposed model in addition to five other DL models using five test lists. Figure 8 presents a segmentation of the COVID-19 infection, which demonstrates that our technique works better than the baseline methods. The output of the approach that was proposed is quite close to ground truth. The detailed results are presented in Table 5.

From Table 5, it has been observed that UNET achieves DSC of 84.19%, and ACU of 84.15%. Similarly, UNET++ achieves the DSC of 86.01%. The proposed model achieves the DSC of 95.50% which is 9.49% and 11.31% superior to UNET++ and UNET, respectively.

### 4.3. Analysis of FL Security

**Figure 9:**
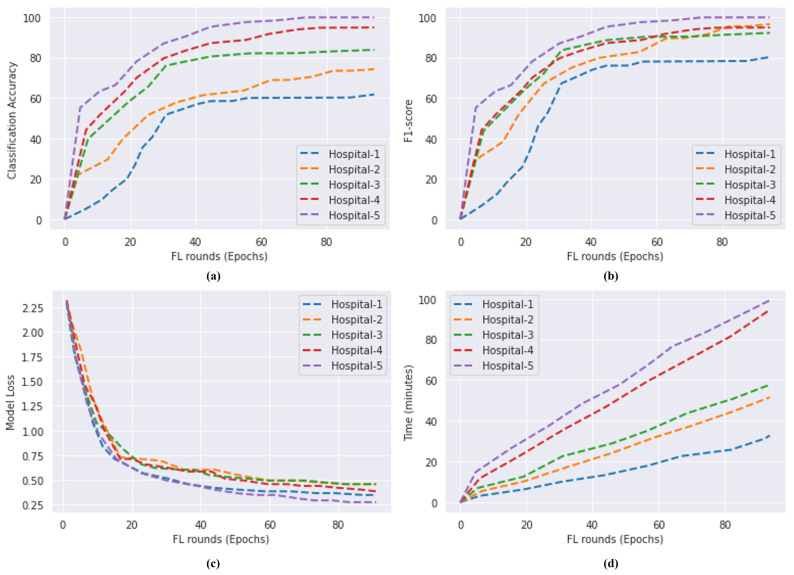
A result achieved by the proposed blockchain-based FL model. (**a**) Accuracy of the proposed model on COVID-19 CT scan dataset for five different hospitals. (**b**) F1-score of the proposed model; (**c**) model loss on COVID-19 CT scan dataset five different hospitals. (**d**) Time of COVID-19 CT scan dataset for five different hospitals using FL.

The data came from a wide variety of places, including hospitals equipped with a wide range of different sorts of CT scan devices. To conduct an accurate performance analysis of FL, the datasets have been split among five different hospitals. As a result of this agreement, several hospitals will be able to share data and take advantage of FL. The performance of our proposed blockchain-based FL model is shown in Figure 9a to show how it changed when the hospitals or providers were varied. The use of a wide variety of data sources produces improved results. The F1-score achieved by the proposed model after distributing the data into five different hospitals is shown in Figure 9b. The convergence of model loss is seen in Figure 9c. The amount of time required to run models that make use of the FL framework is shown in Figure 9d. Because samples obtained from multiple hospitals are not the same, the accuracy does not fluctuate gradually as illustrated in Figure 9a. The number of patients or CT scan slices has a direct bearing on the level of accuracy achieved. A similar approach may be used with the model loss as well. The local models are combined into one global model, and the local models are trained using standardized data from each hospital. The success of the collaborative approach is affected by the total number of hospitals involved. Table 4 compares the FL model with the local model. The local model is trained using the whole dataset, whereas the FL model is instructed by the local models. Figure 9a,b indicate that as the number of data sources increases, performance improves considerably. Nonetheless, FL does not affect accuracy, and it accomplishes data sharing while maintaining anonymity.

In addition, the distinctions in privacy analysis refer to a methodical process that enables hospitals to gain insight from the vast majority of data while simultaneously ensuring that these findings do not enable any individual to differentiate between the data or re-identify the data. In other words, the process enables hospitals to gain insight from the vast majority of data while protecting the privacy of individuals. Equation (18), on the other hand, is used to guarantee that the data’s integrity is maintained by extracting the value that is included inside the data. The decentralized trust system provided by BCT makes it possible for everything to work automatically according to a preset program, which results in increased data security. A stringent collection of algorithms is used by the decentralized BCT, which allows it to give an assurance that the data is authentic, accurate, transparent, traceable, and tamper-proof because it employs a demanding set of algorithms. The suppliers of the data are responsible for their information and can make any required adjustments. The database that the blockchain stores is then updated with real data, together with the digital signature of the owner. Using the smart contract, the owner of the data has the power to administer and modify the policy of the database. This permission is granted to the owner. A large number of cryptographic protocols are implemented inside the blockchain to guarantee the data of its users remains private.

### 4.4. Comparison with State-of-the-Art Methods

COVID-19 has been the focus of several studies, such as [10,11,12,13,14,20,25]; however, these approaches do not take into consideration data sharing to train a better prediction model. However, certain algorithms combine GAN with data augmentation (DA) to produce faked visuals. The reliability of such methods cannot be guaranteed when it comes to medical photos. The examination of the data is difficult since there is only a little quantity of patient information [70]. Our proposed method gathers a significant number of CT scans from five different databases and uses this information to develop a more accurate prediction model. To begin, we examine the current state-of-the-art research about the DL models shown in Table 6.

In addition, we examine FL alongside pre-trained models such as VGG-16 and 19, ResNet, MobileNet, DenseNet, and Capsule Network, amongst others. According to the findings, the accuracy is comparable regardless of whether the local model is trained with the entire dataset or whether the data is divided among hospitals and the model weights are combined using blockchain-based FL. In the last part of this article, we contrast our method with data-sharing tactics that are based on BCT. A DL and blockchain-based solution to the sharing of medical pictures was presented in the study [73]. The fundamental shortcoming of the model is that it is not based on FL and does not aggregate neural network weights across the blockchain. In addition, [82,83] presents a framework for FL; however, they only investigate the possibility of sharing vehicle data. The global model is instructed by our proposed framework to collect data from a variety of hospitals, and it is instructed to operate as a collaborative global model.

### 4.5. Computational Cost

**Figure 10:**
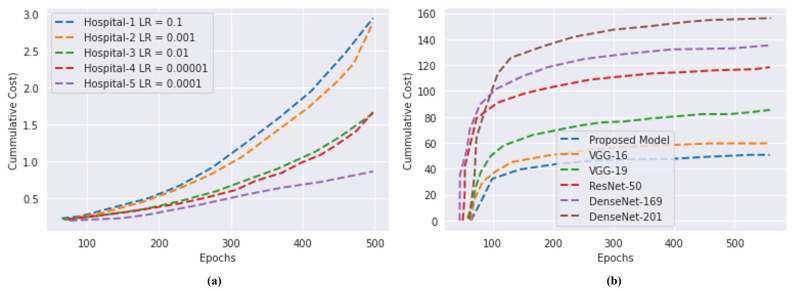
Comparison of the computational cost of the models. (**a**) Proposed blockchain-based FL cost. (**b**) Computational cost comparison with five DL and FL.

A comparison is made between the local various DL learning models and the blockchain-based FL (CapsNet with IELMs) that has been suggested. Despite this, federated architectures based on blockchains provide a high level of both security and privacy. Figure 10a indicates that the communication load increases as the number of hospitals or transactions increases, which in turn causes the operational costs to increase. Furthermore, as can be shown in Figure 10b, the computational cost of our proposed system is less than that of the various traditional DL models.

### 4.6. Discussions

According to the findings of this research, it is feasible to aggregate COVID-19 data utilizing blockchain-based FL across participating centers, while still ensuring patient anonymity while employing this approach. When there is little time to set up intricate data-sharing agreements across organizations or even nations, this decentralized training strategy might be an essential facilitator for scaling AI-based solutions during a pandemic. However, the medical imaging scenario is more complicated and entails unique challenges (such as high-dimensional data [89,90,91,92] and imbalanced cohort sizes [91]), which exert unexplored influences on currently used FL techniques [81]. Recently, the use of FL has been shown in other fields such as edge computing [84,93] (for example, digital devices [81]). To the best of our knowledge, this is the prior research that demonstrates both the practicability and effectiveness of blockchain-based FL for COVID-19 image interpretation where collaborative effort is especially advantageous in times of global crisis. Our observations suggest that blockchain-based FL improved generalization performance overall with single-site models and their ensemble, demonstrating successful decentralized optimization with diverse training data distributions.

In this study, we have applied the blockchain-based FL technique to our proposed CapsNet with the IELMs model. The proposed model is compared with five different DL models in terms of different metrics such as ACU, REC, SPF, F1-score, and PRE. Additionally, we have used the CT scan images to diagnose COVID-19 with these models. Moreover, we collected the CT scan images from five different publicly [56,57,58,59,60,61] available sources. Each CT scan images dataset has been categorized into five different clients while each client represents each hospital. Different CT scans contained different types of resolutions because different CT scan scanners (such as Brilliance ICT, Samatom definition Edge, and Brilliance 16P CT) were used to create the images. Two techniques, signal normalization and spatial normalization, have been used to normalize the data. Afterward, we used the SegsCap to segment the COVID-19-infected portions in the CT scan images. The segmented portions of COVID-19 were used for training of CapsNet with IELMs and five different models such as VGG-16, VGG-19, ResNet-101, DenseNet-169, and DenseNet-201. The proposed model achieved 98.99% accuracy, which is more than other DL models. The proposed model has been used in collaboration with a blockchain-based FL framework to divide in different hospitals, which has been represented in Figure 4. We have also compared the proposed model with the state-of-the-art classifiers, which can be seen in Table 6. The study of experimental results with modern state-of-the-art methodology demonstrates that the suggested model for identifying COVID-19 using CT scans has contributed significantly to the clinical expert’s assistance. An ensemble of 3D-ResNet networks with a channel-based attention module was developed by Kuzdeuov et al. [82], and they obtained a classification accuracy of 93.30% while attempting to identify COVID-19. When categorizing the COVID-19 CT scan images, Wang et al. [83] constructed a unique CNN model that is ensembled with other ML classifiers to obtain optimal results. They succeeded in achieving an 82.92% precision rate. A 2D-CNN model was created by Jin et al. [84] to categorize COVID-19, as well as pneumonia including viral and bacterial infections, and normal pictures. Their model was accurate 94.41% of the time. The study that was given by Chen et al. [91] produced a classification performance of 95.20% in terms of accuracy for the UNET++ with CNN model when applied to CT scan pictures of several chest illnesses. Based on chest CT images, Li et al. [92] built a CNN-based pre-trained model that they called ResNet-50. This model is capable of automatically diagnosing COVID-19 as well as pneumonia. They had a success rate of 90% when it came to making appropriate diagnoses.

## 5. Conclusions and Future Work

In this study, a system has been built that has the potential to make use of existing data to improve the identification of CT scans, as well as to facilitate the interchange of data across hospitals while preserving patient anonymity. The problem of heterogeneity in data is tackled head-on by the method of data normalization. In addition, COVID-19 patients are recognized by the use of CapsNet with IELMs-based segmentation and classification, as well as a method for collaboratively training a global model through the application of BCT and FL. For training and evaluating the proposed model and five different DL models, extensive experiments were performed on a large number of publicly available COVID-19 CT scan datasets. The CapsNet combined with IELMs achieved the highest level of accuracy, which was 98.99%. The proposed model is adroit since it can learn from data collected from conventional hospital sources. Furthermore, if hospitals continue to share the confidential data that they collect to train a more accurate global model, the proposed model could be able to assist in the identification of COVID-19 patients via the use of lung screening. The limitation of this study is that the proposed model is not suitable for sonography and CXR images in its current state. However, in the future, we will combine blockchain and FL with a deep attention module for the classification of different chest diseases including COVID-19, lung cancer, and tuberculosis using different medical imaging modalities such as CXR, CT scans, and sonography.

## Figures and Tables

**Table 1:** Summary of related work for the identification of COVID-19 by using FL and DL models.

Ref	Models	Objective	Image Type		FL	Outcomes
			CXR	CT		
[37]	EMR CXR AI	To identify the COVID-19-infected patients.	✓	×	✓	AUC = 0.92
[38]	FL + CNN	To detect lung abnormalities that occur due to COVID-19.	×	✓	✓	Specificity = 95.27%
[39]	FL + Semi-supervised Learning	To segment the COVID-19-infected regions of the lungs.	×	✓	✓	Accuracy = 0.71
[40]	Capsule Network	To find the COVID-19 infection in the lungs.	×	✓	✓	Precision = 0.83
[50]	FED-GAN	To identify the COVID-19-infected individuals.	×	✓	✓	Precision = 96.6%
[51]	3D UNET	To segment the infected areas of the lungs.	×	✓	✓	Accuracy = 98.07%
[52]	RF	To predict COVID-19 virus activity	×	✓	✓	Accuracy = 81.80%
[53]	ResNet-18	To find evidence of COVID-19’s existence in the lungs.	✓	✓	✓	Accuracy = 97.78%
[54]	AlexNet	To identify COVID-19 from pneumonia.	×	✓	✓	Accuracy = 96.29%
[55]	Multiple CNN models	To forecast the COVID-19 illness	×	✓	×	Accuracy = 94.00%

**Table 2:** Summary of the five datasets of CT scans of COVID-19 and normal cases.

Datasets	No. of Patients	COVID-19 CT Scans	Normal CT Scans	Image Format	Type of CT Scans	Total Number of CT Scans
Dataset 1 [56]	1000	35,635	9367	DICOM	2D	45,002
Dataset 2 [57]	89	28,395	5611	PNG	3D	34,006
Dataset 3 [58]	NA	1252	1230	PNG	2D	2482
Dataset 4 [59]	1110	125	254	3D CT scans	3D	379
Dataset 5 [60]	216	349	463	PNG	2D	812

**Table 3:** Comparison between CapsNet and traditional ANNs.

Steps	Process	CapsNet	Traditional ANN
1	Output	Vector (V_j_)	Scaler (S_j_)
2	Affine Transformation	${\hat{A}}_{u|h}=T_{u,h},\;A_{u}$	NA
3	Weighted Sum	$S_{a}=\sum_{i}M_{i,a}*\;{\hat{A}}_{u|h}$	$S_{a}=\overset{3}{\underset{i=1}{\sum }}M_{i}X_{i}+C$
4	Activation Function	$S_{K}=\frac{||S_{K}|{|}^{2}}{1+||S_{K}|{|}^{2}}\;\frac{S_{K}}{\left||S_{K}|\right|}$	$I_{L,K}\left(A\right)=F\left(X_{j}\right)$

**Table 4:** Comparison of the proposed model with five DL models.

Models	Nodes	Pre-Trained	ACU	PRE	REC	SPF	F1-Score
VGG-16	MLP	ImageNet	91.75%	91.42%	91.47%	91.29%	91.22%
VGG-19	MLP	ImageNet	92.21%	92.09%	92.12%	92.08%	92.14%
ResNet-101	MLP	ImageNet	94.81%	94.21%	94.32%	94.27%	94.22%
DenseNet-169	MLP	ImageNet	94.99%	95.01%	94.96%	94.97%	95.00%
DenseNet-201	MLP	ImageNet	95.45%	95.54%	95.47%	95.43%	95.48%
Proposed Models	Capsule Network and IELMs	-	98.99%	98.96%	98.97%	98.95%	98.96%

**Table 5:** Comparison of the proposed model with UNET and UNET++ for the segmentation process.

Models	ACU	PRE	REC	SPF	F1-Score	DSC
UNET	84.15%	84.42%	84.99%	84.75%	84.22%	84.19%
UNET++	86.10%	86.99%	86.09%	86.99%	86.02%	86.01%
Proposed Model	95.81%	95.49%	95.32%	95.57%	95.51%	95.50%

**Table 6:** Performance comparison of the proposed model with recent state-of-the-art.

Ref	Models	Disease Classification	Accuracy (%)	Data Sharing	BCT Privacy Protection
[82]	3D-ResNet + Attention	COVID-19, Pneumonia, and Normal	93.30	No	No
[83]	CNN	COVID-19 and Normal	82.90	No	No
[40]	Capsule Network	COVID-19 and Normal	98.0	Yes	Yes
[84]	2D-CNN	COVID-19, Pneumonia, and Normal	94.41	No	No
[85]	2D-CNN	COVID-19, Influenza, and Normal	86.70	No	No
[86]	ResNet-50	COVID-19, Pneumonia, and Normal	86.00	No	No
[87]	UNET++	COVID-19, Pneumonia, and Normal	97.40	No	No
[88]	Deep learning	COVID-19	98.20	Yes	Yes
[89]	UNET and CNN	COVID-19, Pneumonia, and Normal	90.70	No	No
[90]	RF	COVID-19, Pneumonia (bacterial and viral), and Normal	87.90	No	No
[91]	UNET++ and CNN	COVID-19, Pneumonia, and Normal	95.20	No	No
[92]	ResNet-50	COVID-19, Pneumonia, and Normal	90.00	No	No
Ours	CapsNet with IELMs	COVID-19 and Normal	98.99	Yes	Yes

## Data Availability

Not applicable.
